# Helical Tomotherapy for Postmastectomy Radiotherapy after Immediate Left Breast Reconstruction: A Case Study

**DOI:** 10.7759/cureus.1462

**Published:** 2017-07-12

**Authors:** Dominique Mathieu, Nazanin Shahvary, Nicolas Côté, Kerianne Boulva, Léamarie Meloche-Dumas, Toni Vu, Erica Patocskai, Christina Bernier, David Roberge, Israel Fortin

**Affiliations:** 1 Department of Radiation Oncology, Centre hospitalier de l'Université de Montréal (CHUM); 2 Department of Surgery, Centre hospitalier de l'Université de Montréal (CHUM); 3 Department of Surgery, Centre hospitalier de l'Université de Montréal (CHUM); 4 Département De Chirurgie Oncologique, Centre hospitalier de l'Université de Montréal (CHUM); 5 Department of Radiation Oncology, Centre hospitalier de l'Université de Montréal (CHUM)

**Keywords:** locally advanced breast cancer, postmastectomy radiotherapy, helical tomotherapy, immediate breast reconstruction

## Abstract

A 43-year-old premenopausal female presented with a multicentric infiltrating lobular carcinoma of the left breast with axillary nodes metastasis. She underwent modified radical mastectomy with axillary lymph node dissection (level I and II) followed by a mixed autologous latissimus dorsi flap reconstruction with the addition of prosthesis. The final pathological analysis revealed a 6 cm invasive lobular carcinoma pT3N2aM0, grade III/III, estrogen and progesterone positive, human epidermal growth factor receptor 2 (HER2) negative, with 5/16 positive lymph nodes. She received neoadjuvant chemotherapy with doxorubicin and cyclophosphamide followed by paclitaxel. Post-mastectomy radiotherapy with axillary, supraclavicular and internal mammary lymph nodes (IMLN) irradiation was delivered to a dose of 50 Gy/25 fx.

In this case with multiple risk factors for radiation-induced cardiac toxicity (left-sided lesion, internal mammary lymph nodes (IMLN) irradiation), we discuss the role of helical tomotherapy as a treatment alternative to conventional tangential radiotherapy.

## Introduction

Modified radical mastectomy with immediate breast reconstruction (IBR) has become a well-established treatment option for patients with locally advanced breast cancer seeking maximum disease control, improved cosmetic results, greater psychosocial well-being and decreased morbidity as compared to delayed reconstruction surgery. In a subset of females with lymphatic metastasis with high risk for locoregional recurrence, post-mastectomy radiotherapy (PMRT) has been proven to increase both disease-free survival (DFS) and overall survival (OS) rates [1]. However, concerns have been raised by multiple authors regarding radiation-induced cardiac toxicity and worse aesthetic outcomes in IBR patients receiving PMRT.

This case study aims to get a better understanding of the challenges involving adequate coverage of internal mammary lymph nodes (IMLN) in left breast irradiation. With advances in radiotherapy technologies such as helical tomotherapy (HT), adequate and safe PMRT can be offered to most patients after autologous breast reconstruction with excellent outcomes. Informed consent statement was obtained for this study.

## Case presentation

A 43-year-old premenopausal female presented with a palpable mass in the superior medial quadrant (SMQ) of the left breast. Past medical/family histories were unremarkable except for bilateral breast augmentation with retropectoral silicon implants. Ultrasound-guided breast biopsy of two hypoechogenic irregular lesions (45x41 mm SMQ, 22x19 mm inferior medial quadrant) and three axillary lymph nodes revealed infiltrating lobular carcinoma with extracapsular invasion of axillary lymph nodes. Staging positron emission tomography and magnetic resonance imaging confirmed a multicentric tumor with axillary nodal metastases.

The patient underwent an oncological resection of the left breast with modified radical mastectomy and axillary lymph node dissection of levels I and II. An elliptical incision was made centered on the SMQ. The overlying skin, as well as the areola and the nipple which was subject to a prior biopsy, were resected. The borders of dissection extended superiorly to the clavicle, inferiorly to the rectus abdominis, medially to the sternum and laterally to the latissimus dorsi (LD). Immediate autologous breast reconstruction, which consisted of mobilization of the LD and insertion of prosthesis under the flap was performed subsequently by the plastic surgeon. Postoperatively, the patient developed a hematoma at the LD donor site that required antibiotics and drainage. One month after IBR, the patient underwent a second operation with the purpose of lysing thoracic adherences and breast nipple reconstruction.

The final pathological analysis revealed a 6 cm invasive pleomorphic lobular carcinoma stage pIIIA (pT3N2aM0). Scarff-Bloom-Richardson and Elston-Ellis grade was III/III (tubular score 3/3, nuclear score 3/3, mitotic score 2/3) with lymphovascular invasion. A single pathology slide revealed a close medial margin (< 1mm). Immunohistochemistry showed positive staining for estrogen receptor and progesterone receptor. Human epithelial growth factor receptor 2 (HER-2) was negative. Five lymph nodes out of 16 were positive and there was focal extracapsular involvement.

The patient had adjuvant chemotherapy as per the National Surgical Adjuvant Breast and Bowel Project (NSABP) protocol B47 trial and randomized to doxorubicin 60mg/m2 IV + cyclophosphamide 600mg/m2 IV every three weeks for four cycles followed by paclitaxel 80mg/m2 IV weekly for 12 doses (Arm 2). Hormone therapy was also provided with tamoxifen 20 mg daily. 

**Figure 1:**
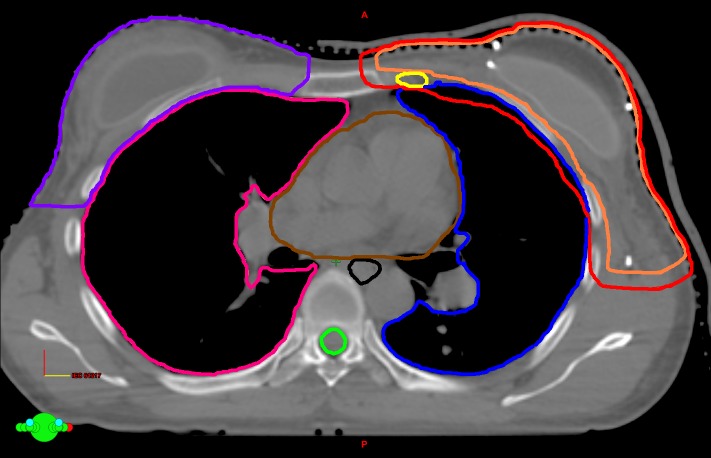
Target volumes and organ at risks (OARs) contoured on the axial slice of the planning computed tomography. Clinical target volume (orange), heart (brown), left lung (blue), internal mammary nodes (yellow), planning target volume (red) oesophagus (black), right breast (purple), right lung (pink), spinal cord (green)

She received PMRT with axillary, supraclavicular and IMLN irradiation. Treatment was delivered with HT to a conventional dose of 50 Gy/25 fx, 5 fx/week, with daily 2 mm chest wall bolus applied to the skin. A free-breathing (FB) planning computed tomography (CT) scan was acquired in the same supine treatment position with both arms abducted above the head and the left breast immobilized with a thermoplastic shell. A radio-opaque wire was placed on the reconstructed breast for clinical target volume (CTV) delineation and planning target volume (PTV) was defined by adding a three-dimensional (3D) uniform expansion of 7 mm around the CTV. The organ at risks (OARs) and target volume contours was performed as per Radiation Therapy Oncology Group (RTOG) 1304 guidelines as shown in Figure 1. The treatment plan was optimized such that the prescribed dose covered at least 95% of the PTV. By the end of radiotherapy treatment, the patient suffered from grade two radiation dermatitis and grade one esophagitis respectively treated with topical application of silver sulfadiazine cream and mouth gargle of a solution containing an aluminum hydroxide and magnesium hydroxide based antacid, lidocaine 2% and diphenhydramine.

**Figure 2:**
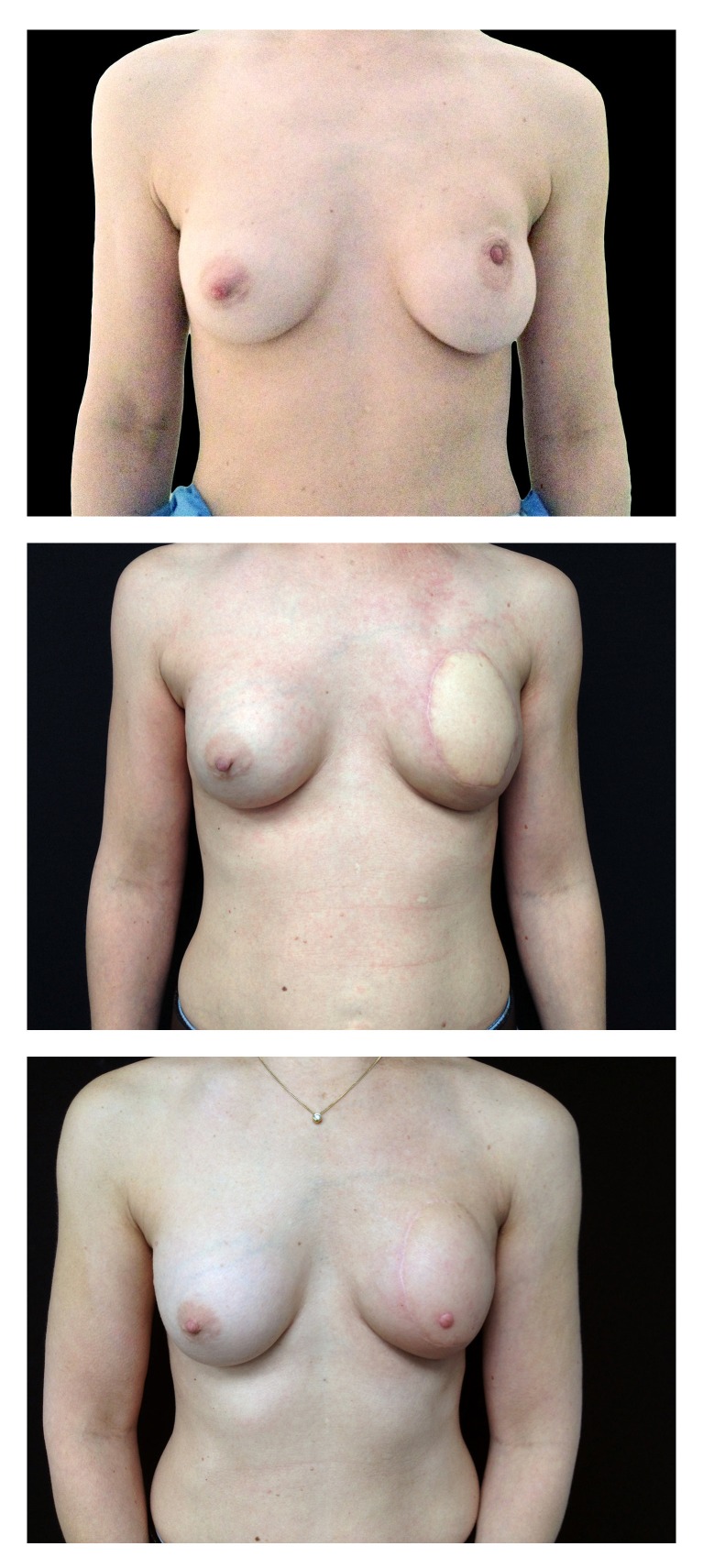
Representative images of immediate breast reconstruction in the setting of postmastectomy radiation, published with permission from the patient. Preoperative (top), post-autologous latissimus dorsi flap reconstruction with addition of prosthesis (middle), post nipple reconstruction (bottom)

Post-therapy follow-up was performed by members of the treatment team and included physical examination every six months, the mammogram every year and serial assessments of cardiac function with radionuclide ventriculography. At three-year follow-up, the patient is disease free and no cardiac toxicity was observed. Immediate breast reconstruction with autologous tissue has provided excellent cosmetic outcome as shown in Figure 2.

## Discussion

Recent randomized trials addressing PMRT in locally advanced breast cancer show clear survival benefits in patients treated with appropriate surgical techniques (complete axillary lymph node dissection) and modern adjuvant systemic therapies. In the Early Breast Cancer Trialists’ Collaborative Group (EBCTCG) meta-analysis, 78 randomized trials, including the Danish Breast Cancer Cooperative Group (DBCG) 82b and 82c as well as the Columbia PMRT trials, were examined for a total of 42,000 females treated [1]. After mastectomy with axillary clearance in node positive patients, PMRT reduced 15-year breast cancer mortality and all-cause mortality from 60.1% to 54.7% and 72.3% to 68.8% respectively (all significant). Although comprehensive PMRT with irradiation of regional lymph nodes is well-established for this subgroup of female, the criteria for patient selection who require IMLN inclusion in the treatment fields remain controversial. Historically, no significant survival benefit has emerged from numerous randomized trials addressing the role of IMLN surgical resection [2-3]. However, recent literature shows a slight but significant improvement in recurrence rate and breast cancer mortality in node positive patients receiving radiotherapy after mastectomy and axillary dissection [4]. In our practice, patients with ≥ 4 positive axillary nodes or those with clinical or pathological disease involvement usually receive ipsilateral IMLN irradiation.

Left-sided whole breast or chest wall radiation therapy carries a long-term risk of cardiac toxicity. The probability of major coronary events (myocardial infarction, coronary revascularization, and death from ischemic heart disease) increases linearly with the mean dose to the heart and left anterior descending artery (LAD), with no minimum threshold for risk [5]. Although reduction of the irradiated cardiac volume can be accomplished through personalized field shaping using multileaf collimator, tangential intensity-modulated radiotherapy (IMRT) often presents suboptimal heart doses in left-sided breast cancer patients. In our institution, cardiac avoidance is rigorously performed through a radiotherapy treatment allocation algorithm. Alternative techniques to tangential radiotherapy such as deep inspiration breath-holding (DIBH) and HT are preferred in all left-sided breast cancer patients. During DIBH, lung inflation creates a separation between the heart and chest wall, moving the cardiac silhouette outside of the tangential fields with subsequent mean heart dose reduction. Helical tomotherapy enables coverage of complex volumes with the excellent conformity of the dose distribution throughout a rotating gantry with treatment delivery from 360 degrees around the patient.

**Figure 3:**
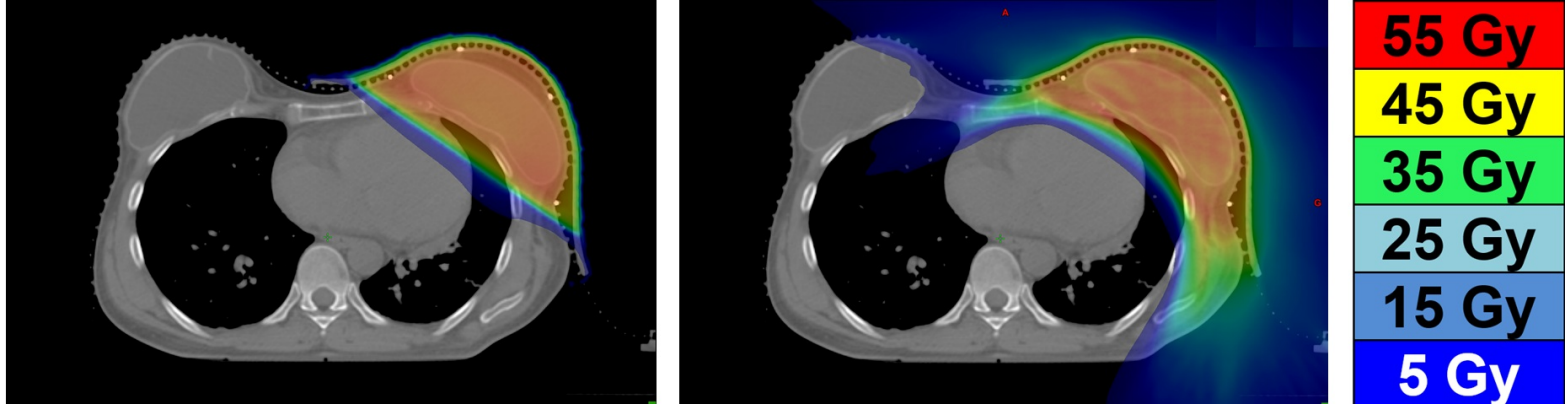
Comparison of prescription isodose color wash of extended tangential radiotherapy (left) and helical tomotherapy (right) plans

In the present case, tangential radiotherapy would have required partially wide tangents for proper IMLN inclusion in the treatment field further exposing the heart to irradiation. Consequently, HT treatment was preferred. Figure 3 shows the dose distribution of partially widened tangential radiotherapy plan versus HT plan, retrospectively optimized to obtain an equivalent 95% coverage of the PTV. In the tangential radiotherapy plan, depth of heart penetration and in-field heart volumes were respectively 1.5 cm and 24 ccs. Compared to tangential results, HT resulted in a reduction of mean heart dose (3.5 vs 3.7 Gy) with more significant effect on the mean dose to left anterior descending (LAD) (22.5 vs 32.7 Gy). As an increase in stenosis of LAD has been reported in left-sided irradiated breast cancer, reduction of doses to coronary arteries is clinically relevant [6]. Helical tomotherapy (HT) delivers a larger dose spread with increased low-dose areas, especially for organs not normally irradiated in tangential radiotherapies, such as the esophagus, the contralateral lung, and breast. To minimize any risk of late secondary malignancy, strategies for OARs preservation are used during treatment planning such as rigorous selection of optimization parameters, field blocking at specific angles and usage of virtual optimization structures for dose constraint. Therefore, both adequate target coverage and optimal organ sparing can be obtained with HT in most patients with strict observance of RTOG dosimetric criteria. Although HT seems to be well tolerated according to early follow-up result and dose-volume histogram analysis, more prospective long-term data are needed to provide hard evidence of the benefit of this technique in terms of reduced toxicity and clinical outcomes. In the presented case, only acute skin toxicity was observed, a common side effect with breast HT with retrospective series reporting a majority of patients with low grade radiodermatitis [7].

Post mastectomy radiotherapy increases the likelihood of capsular contraction, fibrosis, volume loss and delayed wound healing in all types of breast reconstruction. Fewer rates of these complications and increased post mastectomy patients’ satisfaction may be observed in females with immediate autologous reconstruction as compared to implant-based reconstruction [8-9]. Still, preferred surgical approach (myocutaenous flap, expender, implant reconstruction, etc.) may vary among cancer centers and timing of reconstruction in the setting of adjuvant radiation therapy remains a controversial issue. On one hand, IBR offers safe oncological outcomes and may confer a psychological benefit and improvement in self-image [10]. On the other hand, concerns with regards to radiation delivery and loss in reconstruction cosmesis warranted many centers to favour delayed or delayed-immediate reconstruction with placement of a tissue expander at the time of mastectomy when PMRT is administered. Reconstruction selection must be assessed in a multidisciplinary discussion to adopt the surgical approach that best suits the patient’s concerns, risk factors (age, body habitus, obesity, comorbidities, etc.) and treatment team expertise. In the presented case, IBR consisting of a mixed autologous latissimus dorsi (LD) flap reconstruction with the addition of prosthesis was performed to obtain adequate breast volume. The patient completed PMRT eight months post surgery after adjuvant chemotherapy was completed. Despite risk factors of IBR complications (prior breast surgery, lymph node invasion and usage of skin bolus), HT treatment was well tolerated as the patient only developed transient grade 2 dermatitis. The patient and her oncology team were pleased with the cosmetic outcome.

## Conclusions

Alternative treatment options to tangential intensity-modulated radiotherapy (IMRT) such as helical tomotherapy (HT) should be offered to females with left-sided breast cancer or those requiring internal mammary lymph nodes (IMLN) incorporation in treatment field to avoid delayed radiation-induced cardiac toxicity. With advanced treatment technologies, safe and adequate adjuvant post-mastectomy radiotherapy (PMRT) can be offered to females getting immediate breast reconstruction (IBR) surgery with excellent outcomes.
